# Partner Choice in Spontaneous Mitotic Recombination in Wild Type and Homologous Recombination Mutants of *Candida albicans*

**DOI:** 10.1534/g3.119.400516

**Published:** 2019-09-19

**Authors:** Alberto Bellido, Toni Ciudad, Belén Hermosa, Encarnación Andaluz, Anja Forche, Germán Larriba

**Affiliations:** * Department of Biomedical Sciences, Microbiology, University of Extremadura, Avda de Elvas s/n, 06006 Badajoz, Spain and; † Department of Biology, Bowdoin College, Brunswick, ME

**Keywords:** *Candida albicans*, single-strand annealing, inter-homolog recombination, Rad52, Rad51, Rad59

## Abstract

*Candida albicans*, the most common fungal pathogen, is a diploid with a genome that is rich in repeats and has high levels of heterozygosity. To study the role of different recombination pathways on direct-repeat recombination, we replaced either allele of the *RAD52* gene (Chr6) with the *URA*-blaster cassette (*hisG-URA3-hisG*), measured rates of *URA3* loss as resistance to 5-fluoroorotic acid (5FOA^R^) and used CHEF Southern hybridization and SNP-RFLP analysis to identify recombination mechanisms and their frequency in wildtype and recombination mutants. FOA^R^ rates varied little across different strain backgrounds. In contrast, the type and frequency of mechanisms underlying direct repeat recombination varied greatly. For example, wildtype, *rad59* and *lig4* strains all displayed a bias for *URA3* loss via pop-out/deletion *vs.* inter-homolog recombination and this bias was reduced in *rad51* mutants. In addition, in *rad51*-derived 5FOA^R^ strains direct repeat recombination was associated with ectopic translocation (5%), chromosome loss/truncation (14%) and inter-homolog recombination (6%). In the absence of *RAD52*, *URA3* loss was mostly due to chromosome loss and truncation (80–90%), and the bias of retained allele frequency points to the presence of a recessive lethal allele on Chr6B. However, a few single-strand annealing (SSA)-like events were identified and these were independent of either Rad59 or Lig4. Finally, the specific sizes of Chr6 truncations suggest that the inserted URA-blaster could represent a fragile site.

During normal cell proliferation, spontaneous DNA lesions arise at measurable rates and their frequency is significantly increased by the presence of environmental compounds generally referred to as genotoxins. For instance, humans are estimated to generate up to10^5^ mutations/cell/day (Hoeijmakers 2009). To repair DNA lesions, cells have evolved a variety of mechanisms that remove damage and accurately restore genetic information (Boiteux and Jinks-Robertson 2013; Wyrick and Roberts 2015). However, repair may also cause genomic rearrangements whose location and frequency are influenced by the genome structure, particularly by the presence of repetitive elements (Chan and Kolodner 2011). Repeated copies of DNA segments are potential targets for homologous recombination (HR) if resection of double strand breaks (DSB) exposes the complementary sequences (Aguilera *et al.* 2000; Prado *et al.* 2003; Heyer *et al.* 2010; Symington *et al.* 2014).

Single-strand annealing (SSA) plays a major role in direct-repeat recombination resulting in the loss of one repeat and the intervening sequence (Klein *et al.* 2019). Studies in haploid *Saccharomyces cerevisiae* (*S. cerevisiae*) strains on DSB-induced repeat recombination have shown that SSA was dependent on the annealing activity of Rad52 for repeat length of 1-2 kb (Rudin and Haber 1988; Fishman-Lobell *et al.* 1992; Sugawara and Haber 1992; Jablonovich *et al.* 1999) but not when repeats were much larger (*e.g.*, *CUP1* gene or rRNA gene arrays) (Ozenberger and Roeder 1991). Additional work revealed that this process was significantly impaired in the absence of *RAD59*, a *RAD52* paralog, especially when the direct repeats were short (40-fold for 205 bp repeats) (Petukhova *et al.* 1999; Sugawara *et al.* 2000; Davis and Symington 2001, 2003; Wu *et al.* 2006; Pannunzio *et al.* 2008). In the presence of Rad52 and Rad59, DSB-induced SSA utilized repeats as short as 29 bp and showed linear dependency on the length of homologous repeats up to 415 bp (Ivanov *et al.* 1996).

Non-DSB direct-repeat recombination (spontaneous) via SSA-like mechanisms can also lead to loss of one repeat plus the intervening sequence. In *S. cerevisiae*, the rate of spontaneous direct-repeat recombination (not DSB-induced) was directly proportional to the substrate length and the minimal repeat length for efficient recombination was 285 bp; some recombination was detected for 80 bp repeats but not for 37 bp repeats (Jinks-Robertson *et al.* 1993). This suggests the existence of specific differences between DSB-induced and spontaneous direct-repeat recombination via SSA. Importantly, SSA does not require strand invasion and is therefore independent of Rad51 (and its paralogs Rad55 and Rad57) (Ivanov *et al.* 1996; Jablonovich *et al.* 1999; Pannunzio *et al.* 2008).

The genome of *C. albicans*, the most common fungal pathogen, is particularly rich in direct repeats (Braun *et al.* 2005; Smichd *et al.* 2012; Todd *et al.* 2019). Not much is known in *C. albicans* about the recombination pathways involved in repeat number alteration and the potential consequences for overall genome structure and host-fungus interactions. It is believed that repeat number alterations are caused by replication slippage and recombination and may provide an evolutionary advantage in fluctuating environments thereby providing the population with a selection of proteins with different properties. Not only may these mechanisms alter repeat numbers and generating novel alleles of a specific ORF, recombination between repeats of two genes from the same family (*i.e.*, *agglutinin-like* (*ALS*) sequence gene family in *C. albicans*) could lead to chimera formation, which may be endowed with novel properties advantageous for survival in the host (Zhang *et al.* 2003; Zhao *et al.* 2011). Several studies have shown that repeats within coding regions of genes may have functional roles. For example, the repeat copy number in *ALS5* directly affects adhesion to fibronectin (Rauceo *et al.* 2006). Repeats of Hwp1, Pir1 and Eap1 are important in adhesion to buccal epithelial cells (Staab *et al.* 2004), protein localization (Sumita *et al.* 2005), and positioning of binding sites to several materials and cells, respectively (Li and Palecek 2008). Furthermore, repeat length variation in cell wall-associated proteins may contribute to the overall antigenic variation in *C. albicans*, which in turn aids in adaptation to and evasion from the host (Verstrepen and Fink 2009; Zhang *et al.* 2010; Zhao *et al.* 2011; Zhou *et al.* 2017).

Here, we took advantage of the URA-Blaster cassette which consists of the *URA3* gene of *C. albicans* flanked by 1.1 kb *hisG* direct repeats (Alani *et al.* 1987; Fonzi and Irwin 1993). To study direct-repeat recombination and to test for allele-specific effects, we replaced each allele of *RAD52* (located on the left arm of chromosome 6 (Chr6) with this cassette, measure rates of *URA3* loss as resistance to 5-fluoroorotic acid (5FOA^R^), and then analyzed 5FOA^R^ derivatives by CHEF Southern and SNP-RFLP to determine the underlying genetic events and associated mechanisms (Forche *et al.* 2011). To assess the role of genes important for homologous recombination of direct repeats, we performed the same analyses in strains lacking *RAD52*, *RAD51*, *RAD59*, and *LIG4*. We found that *URA3* loss in wild type, *rad59*, and *lig4* backgrounds mostly resulted from *URA3* pop-outs and to a lesser degree from interhomolog recombination. This bias was maintained in *rad51* strains although with a significant reduction in the frequency of *URA3* pop-outs compared to wild type. In *rad51* 5FOA^R^ derivatives additional *URA3* loss mechanisms were identified including chromosome loss and truncation as well as ectopic translocations. Interestingly, *rad52* 5FOA^R^ derivatives underwent chromosome loss or truncation 85% of the time with interhomolog recombination being absent. The remaining *URA3* loss events resulted from SSA-like mechanisms, which were independent of Rad59 and Lig4. As a collateral and unexpected finding, our results support the possibility that the insertion of the *URA*-blaster into the genome may have resulted in the generation of a slow replication zone and/or fragile site.

## Materials and Methods

### C. albicans strains used in this study

**Figure 1:**
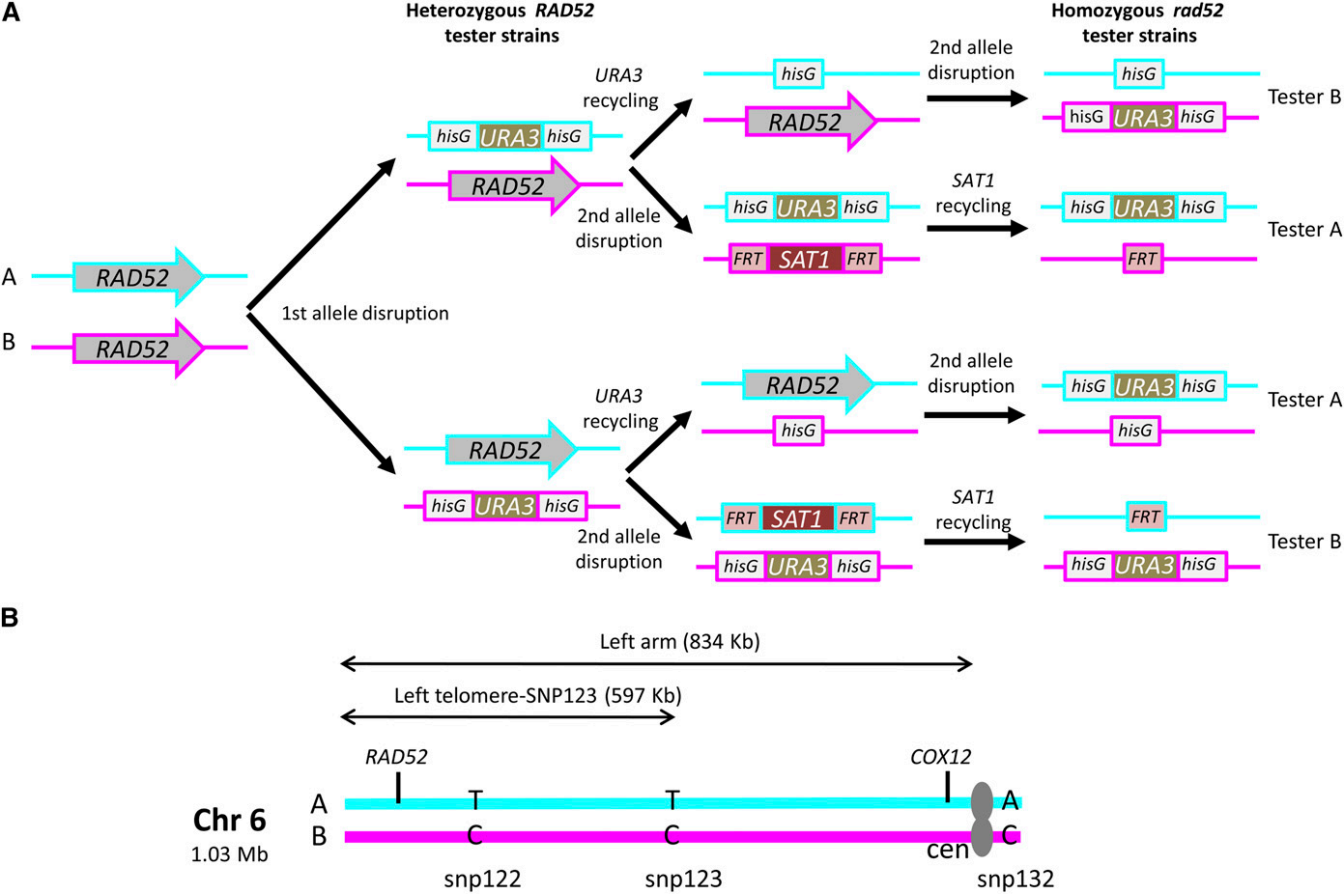
Diagrams. (A) Approach used in this work to generate tester strains with the hisG-*URA3*-hisG cassette either on allele A (cyan) or allele B (magenta) at the *RAD52* locus on Chr6L. (B) Chromosome 6 homologs A (cyan) and B (magenta) and location of SNPs markers 122, 123, (left arm), including their distances from the left telomere, *CEN6 (*cen), and SNP marker 132 (right arm). In panel (A) the alternative allele in *rad52* null strains was either a *hisG* fragment or the FRT site resulting from the eventual excision of the SAT1 cassette, as indicated.

Single and double mutant strains used in this work were generated from strain CAI4, a Ura^-^ derivative of the reference strain SC5314 (Gillum *et al.* 1984), by disrupting the indicated allele with the *hisG-URA3-hisG* cassette flanked by promoter and terminator regions of the target gene (Table S1). Transformants were verified by PCR and/or Southern blot analyses as previously described (Ciudad *et al.* 2004; Bellido *et al.* 2015). To isolate 5FOA^R^ derivatives, a single colony from the indicated genetic background was re-isolated on an YPD plate and then streaked on a new YPD plate supplemented with 0.1% (w/v) 5FOA and 25 µg ml^-1^ uridine, since *C. albicans ura3* mutants are fed with uridine. To disrupt *RAD52* with the SAT1-flipper cassette, the upstream and downstream regions of the *RAD52* ORF were PCR-amplified from genomic DNA of strain CAF2-1, using oligonucleotides RAD52F-*Apa*I/RAD52R-XhoI and RAD52F-*Sac*II/RAD52R-*Sac*I respectively (Fig. S1 and Table S2). Amplified fragments were cloned in pSFS2A plasmid flanking the SAT1-flipper cassette. The disruption cassette was released by digestion with *Apa*I and *Sac*I and transformed into the indicated hemizygous strains *RAD52*/*rad5*2∆::*hisG-URA3-hisG* (Figure 1) using a MicroPulser Electroporator system (Bio-Rad) (Ciudad *et al.* 2016). Nourseothricin-resistant (Nou^R^) colonies were selected on YPD plates supplemented with 200 µg/ml nourseothricin. Several transformants were initially selected based on their thorny colonies and filamentous cell morphology, two phenotypes of null *rad52* strains (Andaluz *et al.* 2006) and then PCR verified for both integration of the SAT1-Flipper cassette in the *RAD52* locus (oligonucleotides SAT1F-Flip/RAD52R) and absence of any residual *RAD52* allele (oligonucleotides RAD52-IF/RAD52-IR). *SAT1* loss was induced by overnight growth in liquid YPM (2% maltose, 1% yeast extract, 2% bactopeptone) (Reuss *et al.* 2004). The resulting nourseothricin-sensitive (Nou^S^) derivatives were selected as small colonies on YPD plates supplemented with 20 µg/ml nourseothricin. They were verified by PCR for *SAT1* loss (oligonucleotides RAD52-F and RAD52-R). These strains carry the *rad52*::*FRT* allele (FRT strains) (Fig. S1).

To determine *URA3* loss rates in the presence of both *RAD52* alleles we used the *SHE9/she*::*hisG-URA3-hisG* reporter. *SHE9*, located in Chr2L (coordinates 615,050 – 616,624), is a non-essential gene whose null homozygous disruptant (Fig. S2) does not show any obvious phenotype (Andaluz *et al.* 2001b) (our unpublished results).

### DNA extraction and analysis

Extraction of genomic DNA, preparation of chromosomes, and CHEF Southern hybridization have been described (Andaluz *et al.* 2011). Two different PFGE protocols were used. In the first protocol (short run), all chromosomes were separated. The second protocol separates both homologs of Chr7 and, in some strains, of Chr6 (Andaluz *et al.* 2011). To test for the presence of one or both homologs of Chr6 we used the SNP status (genotype) of multiple markers along chromosomes as proxy. Routine SNP-RFLPs analyses were carried out as described (Forche *et al.* 2009) using the indicated primers (Table S2).

### Generation, verification and characterization of Chr6A and Chr6B tester strains

In the strain background used in this study, Chr6 homologs exhibit size differences sufficient for separation on CHEF gels. Chr6 homolog length polymorphisms can be due to differences in the number of repeats either within the major repeat sequence (MRS) (Chibana and Magee 2009) or within members of the *ALS* family (*ALS6*, *ALS1*, *ALS10*, *ALS5*, and *ALS2*) located on this chromosome (Zhang *et al.* 2003; Zhao *et al.* 2011).

We used strains heterozygous for *RAD52* and *rad52* null strains to generate tester strains with the hisG-*URA3*-hisG construct either replacing *RAD52* on the A or the B homolog (Table S1). CAGL4A and CAGL4.1A are two independent *rad52* Ura^+^ derivatives of the heterozygous parental, CAGL1B (Ciudad *et al.* 2004). We have previously shown that CAGL4A and CAGL4A.1 conserved both homologs of Chr6 (Andaluz *et al.* 2011). To identify the test homolog in CAGL4A and CAGL4A.1, we first performed a physical analysis of Chr6 homologs present in its parental heterozygous strain CAGL1B.1 (Ura^-^). CHEF Southern hybridization with a *COX12* probe confirmed the presence of both Chr6 homologs in CAF2-1, CAI4, and CAGL1B.1, and hybridization with *RAD52* and *hisG* probes localized *RAD52* to the smaller Chr6 homolog (Chr6A) and *rad52*::*hisG* to the larger homolog (Chr6B) (Fig. S3A). In agreement with this, a spontaneous His^-^ derivative (GLH1-7) of a *rad52* strain (TCR2.1.1) disomic for Chr6 only carried the small homolog and was homozygous, haplotype A, for multiple Chr6 SNPs markers (Forche *et al.* 2009; Andaluz *et al.* 2011).

We also took advantage of heterozygosity within the *RAD52* ORF to identify the *RAD52* allele present in each heterozygote using SNP/RFLP. A 793 bp region of the *RAD52* ORF was amplified with primers RAD52_501F and RAD52_1290R (Table S2) and subjected to a restriction digest with *Taq*I. This enzyme cuts twice in allele A (RAD52A) yielding 3 restriction fragments (251 bp, 237 bp, 305 bp) and once in allele B (RAD52B) resulting in 2 restriction fragments (251 bp and 542 bp) (Fig. S3B). As expected, both alleles were detected in strain CAI4 (as well as in parental strains SC5314 and CAF2-1, not shown) whereas CAGL1B was homozygous for *RAD52A* (Fig. S3B). We concluded that during the generation of CAGL4A and CAGL4A.1, the *RAD52B* allele present in the larger Chr6 (Chr6B) of CAI4 strain was disrupted first resulting in the intermediate strain CAGL1B (test chromosome B) (Fig. S3, top). Because of previous findings that the Chr6B allele may harbor recessive lethal alleles (and therefore cannot be lost) (Andaluz *et al.* 2011; Hickman *et al.* 2013; Feri *et al.* 2016), new strains were generated with the URA-Blaster inserted carrying Chr6A as the test chromosome (CAGL1A). These strains were used to generate *rad52*::*hisG* strains with the opposite configuration, *i.e.*, if derived from CAGL1A, test chromosome was Chr6B, or *rad52*::FRT strains that conserved the parental configuration, *i.e.*, if derived from CAGL1A, Chr6A remained as test chromosome (Figure 1, Table S1). All heterozygous and null *RAD52* strains were tested for the presence of both Chr6 homologs by SNP RFLP (SNP122, SNP123 and SNP132) and for the lack of obvious GCR by PFGE (Fig. S4). We concluded that all of them were appropriate for the generation and subsequent genetic analysis of the 5FOA^R^ derivatives.

### Fluctuation test

Strains were streaked to single colonies on YPD and incubated for 2 - 4 days at 30°. At least 10 independent colonies from each strain were resuspended in 100 µl of sterile water. Tenfold dilutions were generated using 10 µl of the initial resuspension and 40 µl of the 10^−4^ dilution were spotted onto YPD plates to determine the total amount of CFUs. The remaining 90 µl of the initial resuspension was spread onto 5FOA plates. Alternatively, fluctuation analysis using twenty overnight (16 h) liquid cultures seeded with single colonies was done as described by Forche *et al.* (2011). Importantly, for wild type strain CAGL1B, *URA3* loss rates (5FOA^R^) were similar for both methods (1.5 × 10^−5^/cell generation for colonies *vs.* 2.5 × 10^−5^/cell generation for liquid cultures). Therefore, fluctuation analyses were carried out using the former protocol. YPD and 5FOA plates were incubated at 30° for 3 days and colonies were counted. *URA3* loss rates were calculated as described (Forche *et al.* 2011).

### Molecular characterization of URA3 loss in 5FOA^R^ derivatives

For most strains, a minimum of 20 5FOA^R^ derivatives per strain background were analyzed. A scheme with the several steps for characterization of the 5FOA^R^ derivatives at the *RAD52* locus is shown in Fig. S4. SNP results are summarized in Table S3.

We used *S. cerevisiae* chromosomal markers to determine the size of SNCs (Argueso *et al.* 2008). The calculated size correlated well with the genotypes of markers snp122 and snp132, which are 832 kb and 545 kb away from the right telomere, respectively. SNCs from strains heterozygous for snp122 should be larger than 832 kb, whereas SNCs from strains homozygous for both snp122 and snp123 should be smaller than 545 kb. Importantly, all strains carrying SNCs were heterozygous for snp123 marker, an indication that no SNC was smaller than 545 kb (Figure 1). Fisher’s exact test was used to determine whether the frequency of different loss mechanisms in the mutant strains *vs.* wild type were significant (p value of < 0.05).

The occurrence of SSA at the *SHE9* locus was investigated by PCR using primers *SHE1* and *SHE2*, which amplify bands of 845 bp and 1171 bp for *SHE9* and *hisG* repeat respectively, whereas the presence of *URA3* in 5FOA^R^ segregants (*URA3* mutational inactivation) locus was verified using primers *SHE1* and *URA3det-R* that amplify band of 1.3 kb (Table S2).

### Data availability

Strains and plasmids are available upon request. The authors state that all data necessary for confirming the conclusions presented in the article are represented fully within the article. Supplemental material available at FigShare: https://doi.org/10.25387/g3.8796686.

## Results

### Experimental system

**Figure 2:**
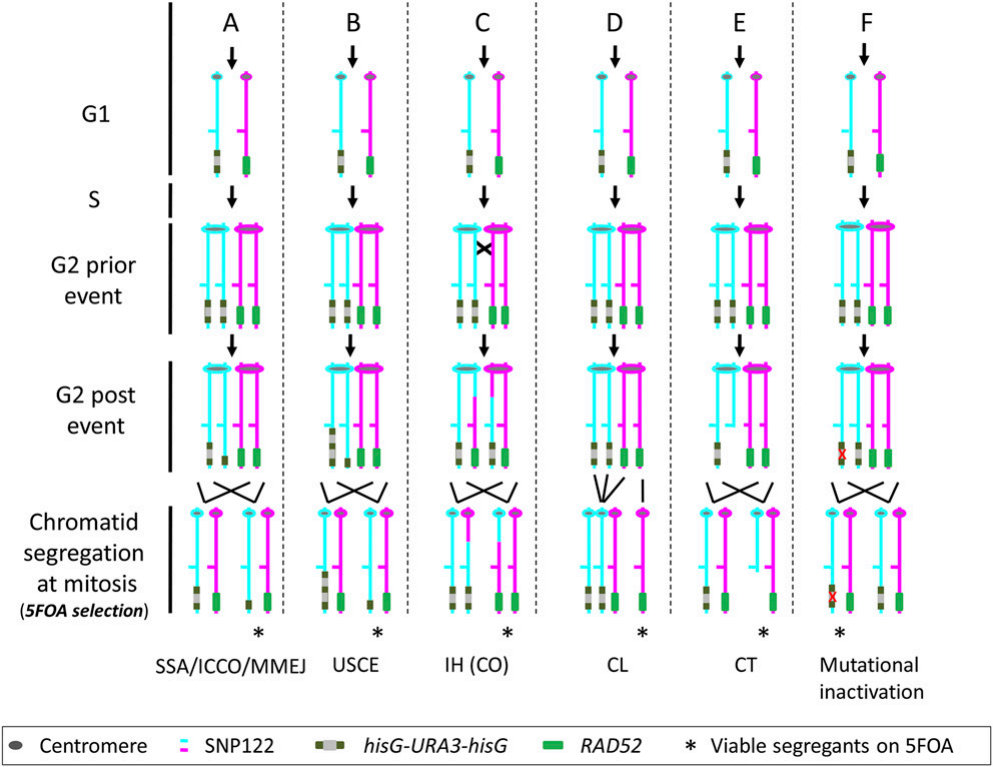
Overview of possible mechanisms leading to inactivation of *URA3* in a *RAD52/rad52*::*hisG-URA3-hisG* strain. Homolog A is shown in cyan and homolog B is shown is magenta. The resulting Ura^-^ derivatives (bottom row) are selected on 5FOA. Viable progeny (only Ura^-^ derivatives can grow on 5-FOA) is indicated with an asterisk. Events can occur in G1 phase of the cell cycle (top row; two homologs) or in G2 (second and third row; both chromatids are maintained together by the centromere), but only G2 events are shown, as follow: pop-out of *URA3* and one copy of the two *hisG* repeats, which can occur via a single-strand annealing-like mechanism involving spontaneous intra-chromatid direct-repeat recombination, intrachromosome or intra-chromatid crossover, or microhomology-mediated end joining (A); unequal sister chromatid exchange (B); inter-homolog recombination including crossover, break-induced replication (BIR) (C), or gene conversion (schematic not shown); ectopic recombination (schematic not shown); chromosome loss (D); chromosome truncation (E); and mutational inactivation of *URA3* (F). Green line: Rad52; black line, hisG; gray line, *URA3*. Note that gene conversion (GC) without crossover at the *RAD52* locus in G1 or G2 is also possible, but the absence of heterozygosities between *RAD52* and the left telomere prevents its detection.

A diagram showing the approach used to generate tester strains with the hisG-*URA3*-hisG cassette is shown in Figure 1A (right side upper branches). We used the *RAD52* locus (Chr6L, left arm of Chr6, coordinates 97,421 to 95,727; see Figure 1B) to determine *URA3* loss rates in Rad52^+^ strains (*RAD52/rad52*::*hisG-URA3-hisG*; wild type in the context of this study) because it also allows the analysis of *rad52* null mutants (*rad52*::*hisG*/*rad52*:*hisG-URA3-hisG*), which are refractory to targeted gene replacement (Ciudad *et al.* 2004). 5FOA^R^ derivatives from Rad52^+^ strains can arise through events shown in Figure 2 (Hiraoka *et al.* 2000; Cauwood *et al.* 2013). The strategy used to identify those events is summarized in Fig. S4. SNP-RFLP analysis allowed us to delimit the genomic region where genetic events responsible for the *RAD52* genotype had occurred. When all SNP markers (snp122, 123 and 132) were homozygous for the same haplotype, the strain was considered having undergone a chromosome loss event (Figure 2D) (Legrand *et al.* 2008; Forche *et al.* 2011). Truncation of the test chromosome (*i.e.*, the chromosome carrying the URA-Blaster) (Figure 2E) results in chromosome fragments detectable by pulsed-field gel electrophoresis (PFGE) and in hemizygosity of *RAD52* and genes between *RAD52* and the left telomere.

### URA3 loss rates in wild type strains and in strains defective for recombination

We determined *URA3* loss rates and associated recombination mechanisms for wild type (*RAD52* het, Ura^+^) and for strains deleted for *RAD59*, *LIG4*, *RAD52* or *RAD51*. To further limit the possibility for single strand annealing to occur, double mutants *rad59 rad52* and *lig4 rad52* were also analyzed. Importantly, for each genetic background (except for *lig4* derivatives), two tester strains carrying the URA-Blaster on either allele of Chr6 at the *RAD52* locus (Chr6A, tester A and Chr6B, tester B) were analyzed (Figure 1 and Table S1).

**Figure 3:**
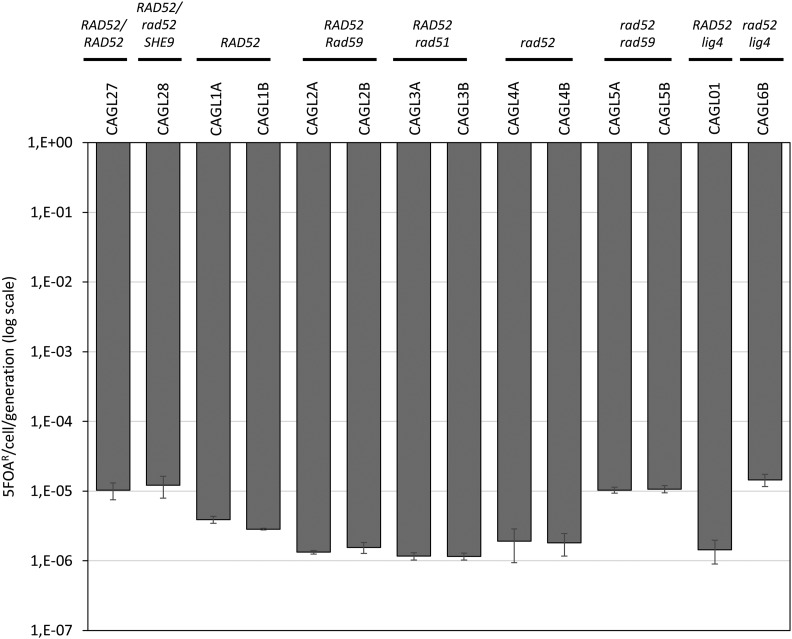
Determination of *URA3* loss rates using fluctuation analysis. Y-axis, Rate of 5FOA resistance/cell/generation. Note: y-axis is a logarithmic scale.

For the *RAD52* het strains *URA3* loss rates for 2 independent assays was 3.9 × 10^−6^/cell generation (STD = 6.8 × 10^−7^) and 2.9 × 10^−6^/cell generation (STD = 5.1 × 10^−7^) respectively (Figures 3 and S5). No significant differences were observed for a *lig4* strain (CAGL01) carrying the URA-Blaster on Chr6A (Figure 3). For all other single deleted strains (Table 1), loss rates were on average 2.5- to threefold lower than those of the wild type equivalent strains, CAGL1A and CAGL1B, respectively. In contrast, compared to the *rad52* single mutant, *URA3* loss rates were higher for *rad52 rad59* (5.fivefold) and *rad52 lig4* (7.eightfold) double mutants (Figure 3) (see Discussion).

**Table 1:** Summary of the events leading to *URA3* loss

Strains*		Genetic events				
	pop-out (*URA3* deletion)	Interhomolog recombination (GC/XO/BIR)	Chromosome loss	Chromosome Truncation	Ectopic Translocation	Mutational Inactivation
CAGL1A (wt, A; 1)	100% (24)	—	—	—	—	—
CAGL1A (wt, A; 2)	90% (18)	10% (2)	—	—	—	—
CAGL1B (wt, B; 1)	92% (22)	8% (2)	—	—	—	—
CAGL1B (wt, B; 2)	90% (19)	10% (1)	—	—	—	—
CAGL2A (*rad59*, A; 1)	85% (17)	10% (3)	—	—	—	—
CAGL2A (*rad59*, A; 2)	95% (19)	5% (1)	—	—	—	—
CAGL2B (*rad59*, B; 1)	83% (20)	12% (3)	5% (1)	—	—	—
CAGL2B (*rad59*, B; 2)	90% (17)	10% (2)	—	—	—	5% (1)
CAGL3A (*rad51*, A; 1)	65% (13)	10% (3)	—	20% (4)	—	—
CAGL3A (*rad51*, A; 2)	73% (22)	3% (1)	—	17% (5)	7% (2)	—
CAGL3B (*rad51*, B; 1)	85% (17)	5% (1)	15% (2)	—	—	—
CAGL3B (*rad51*, B; 2)	67% (20)	3% (1)	27% (8)	—	3% (1)	—
CAGL4A (*rad52*, A)	—	—	—	90% (9)	—	10% (1)
CAGL4.1A (*rad52*, A)	12,5% (1)	—	—	87,5% (7)	—	—
CAGL4B (*rad52*, B)	20% (4)	—	30% (6)	50% (10)	—	—
CAGL5A (*rad59 rad52*, A; 1)	10% (1)	—	—	90% (9)	—	—
CAGL5A (*rad59 rad52*, A; 2)	35% (7)	—	—	65% (13)	—	—
CAGL5B (*rad59 rad52*, B; 1)	—	—	11% (1)	67% (6)	—	22% (2)
CAGL5B (*rad59 rad52*, B; 3)	5% (2)	—	15% (3)	75% (15)	—	—
CAGL6B (*lig4 rad52*, B; 1)	4% (1)	—	46% (11)	50% (12)	—	—
CAGL6B (*lig4 rad52*, B; 2)	10% (3)	—	30% (6)	55% (11)	—	5% (1)
CAGL01 (*lig4*, A; 1)	100% (20)	—	—	—	—	—
CAGL4A-FRT	25% (5)	—	—	75% (15)	—	—
CAGL4B-FRT	10% (2)	—	55% (11)	45% (9)	—	—
CAGL5B**-FRT	15% (3)	—	20% (4)	65% (13)	—	—
CAGL5B-FRT	25% (5)	—	40% (8)	35% (7)	—	—
CAGL6B-FRT	5% (1)	—	55% (11)	40% (8)	—	—
CAGL27	76% (38)	22% (11)	—	—	—	2% (1)
CAGL28	81% (30)	19% (7)	—	—	—	—

For each strain (except CAGL27 and CAGL28) both genotype and test allele (A or B) are indicated. 1 and 2 refers to independent experiments. The number of derivatives analyzed in each experiment is shown in parenthesis. Genetic events are indicated at the top. For strains CAGL1, CAGL2 and CAGL3, 5FOA^R^ derivatives resulting from *URA3* pop-out conserved the wild type *RAD52* allele and the disrupted *rad52*::*hisG-URA3-hisG* allele had been processed to *rad52*::*hisG*. Derivatives resulting from IHR carried only the wild type *RAD52* allele (2 copies). CL and CT were further confirmed by SNP RFLP analysis and CHEF Southerns. nd-not determined. 5FOA^R^ derivatives from FRT strains were screened for SSA, SNPs GRCs. Strain CAGL5B**-FRT was intended to be CAGL5A-FRT (tester A) since it was derived from CAGL2A but behaved as if carrying *RAD52*B as the test allele. It is likely that a reciprocal exchange between both *RAD52* alleles (gene conversion or crossover) occurred at some step during its generation.

### URA3 loss mechanisms in wild type, rad59 and lig4 strains

Next, we examined the nature of genetic alterations associated with *URA* loss using CHEF Southerns and SNP-RFLP analysis (Andaluz *et al.* 2011; Forche *et al.* 2011). PCR of the *RAD52* locus showed that 5FOA^R^ in strains derived from wild type CAGL1A resulted from *URA3* deletion (95.5%, 42/44) and interhomolog recombination (4.5%, 2/44), which was similar for CAGL1B 5FOA^R^ derivatives (Table 1). Karyotype analysis did not identify any gross chromosomal rearrangement (GCR) (Fig. S6) and SNP-RFLP analysis showed homozygosis for snp122 and snp123 for 5FOA^R^ derivatives that resulted from interhomolog recombination and snp132 remained heterozygous (Table S3). This suggests that crossover/BIR between *cen6* and snp123 led to 5FOA^R^ in these derivatives and that both homologs of Chr6 were retained. Taken together, our data for the *RAD52* het strain background show that, irrespective of the Chr6 homolog used as the tester allele, the formation of 5FOA^R^ derivatives is rarely accompanied by chromosome loss or truncation and a strong bias exists for *URA3* deletion *vs.* interhomolog recombination/other events.

Deletion of *RAD59* or *LIG4* in wild type strain background did not alter *URA3* loss mechanisms. For strains CAGL2A and CAGL2B (Rad52 het, *rad59*ΔΔ, Table 1), the *URA3* pop-out bias (88%) was similar to the related wild type strains (Fisher’s exact test, *P* = 0.665, tester allele A, and *P* = 0.314, tester allele B) (Tables 1 and S3). However, three 5FOA^R^ derivatives from strain CAGL2B showed supernumerary chromosomes (SNC) unrelated to Chr6 (two strains are shown in Fig. S7), suggesting that the absence of Rad59 does not alter frequencies of *URA3* pop-out but may cause genetic instability. Similarly, a *lig4* strain (Andaluz *et al.* 2001a; Sinha *et al.* 2016) carrying the URA-Blaster on Chr6A (CAGL01) did not show GCRs, and all 5FOA^R^ derivatives resulted from *URA3* deletion (Tables 1 and S3) suggesting that microhomology mediated end-joining does not contribute either to the observed *URA3* pop-out bias in wild type.

### Chromosome loss and truncation are the prevalent mechanisms leading to 5FOA^R^ in a rad52 strain background

**Figure 4:**
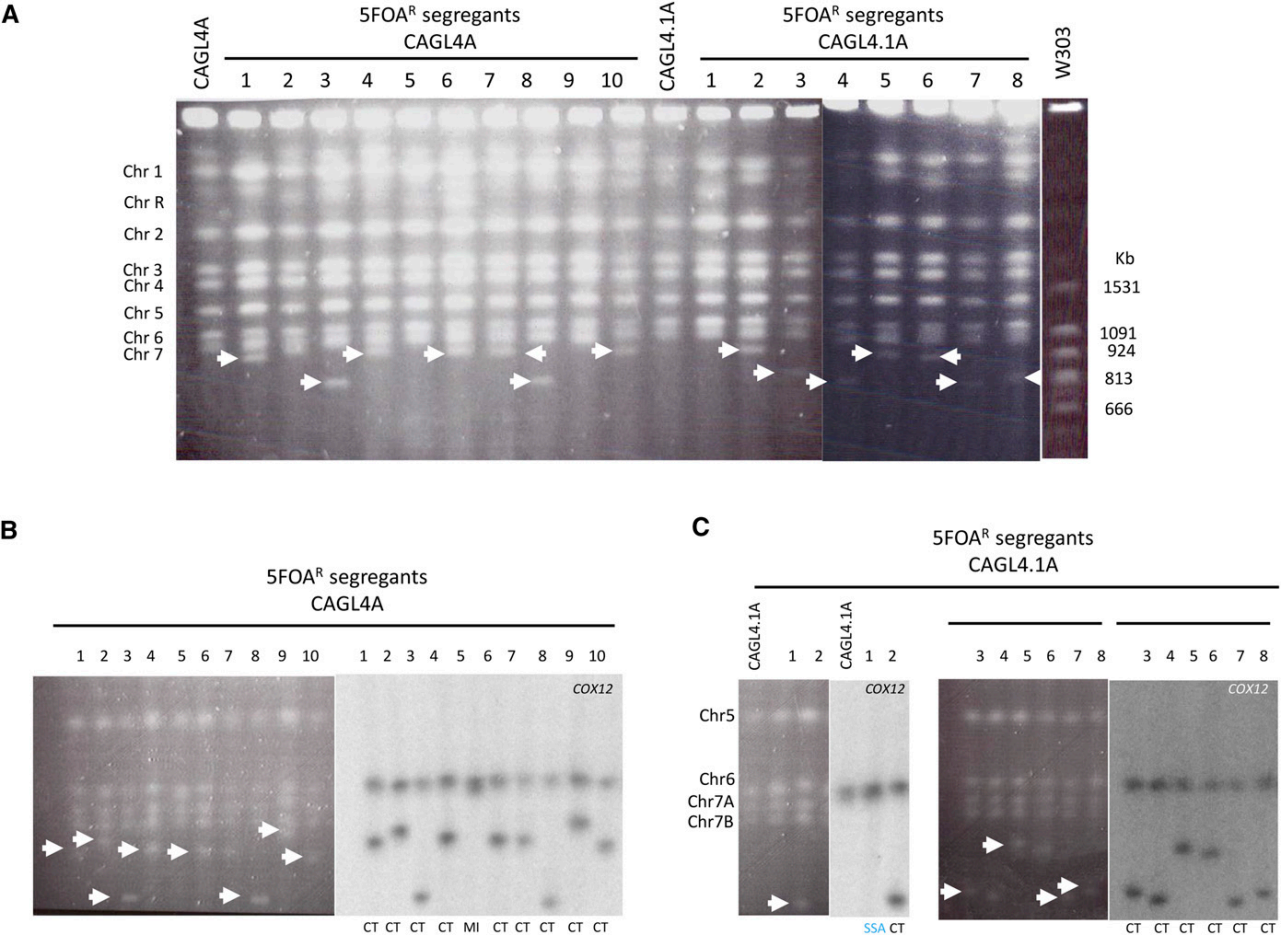
Karyotypes of strains CAGL4A and CAGL4.1A (both test Chr6A) and their 5FOA^R^ derivatives. (A) PFGE gel. B and C) separation of smaller Chrs (5-7) followed by Southern blot hybridization using a *COX12* probe for derivatives of CAGL4A (B) and CAGL4.1A (C). The event determined for each derivative is indicated at the bottom of the CHEF-Southern. SNCs are marked with white arrowheads. Note that most SNCs were larger than 666 kb and the majority had sizes of ∼815 kb (see also Fig. S8), which is in agreement with the genotypes of markers snp122 and snps123 in the 5FOA^R^ derivatives (Fig. 1, Table S3).

Because *C. albicans* strains lacking *RAD52* are intrinsically unstable (Andaluz *et al.* 2011), we first compared two independent, isogenic *rad52* mutants (CAGL4A and CAGL4.1A) (Material and Methods, Table S1) that carry the URA-Blaster on Chr6A. CHEF Southerns indicated that 90% of 5FOA^R^ derivatives of both strains showed novel SNCs between 815 and 945 kb (Figure 4, Table 1) that hybridized to a *COX12* probe (located on Chr6L, Figure 1) suggesting that these were Chr6 truncations. One CAGL4A derivative (CAGL4A-5) acquired a *URA3* loss of function mutation (Figure 4, Table S3) and, quite strikingly, one CAGL4.1A derivative (CAGL4.1A-1) showed a wild type genotype except for the absence of *URA3* (Figure 4A and C) (see also below).

**Figure 5:**
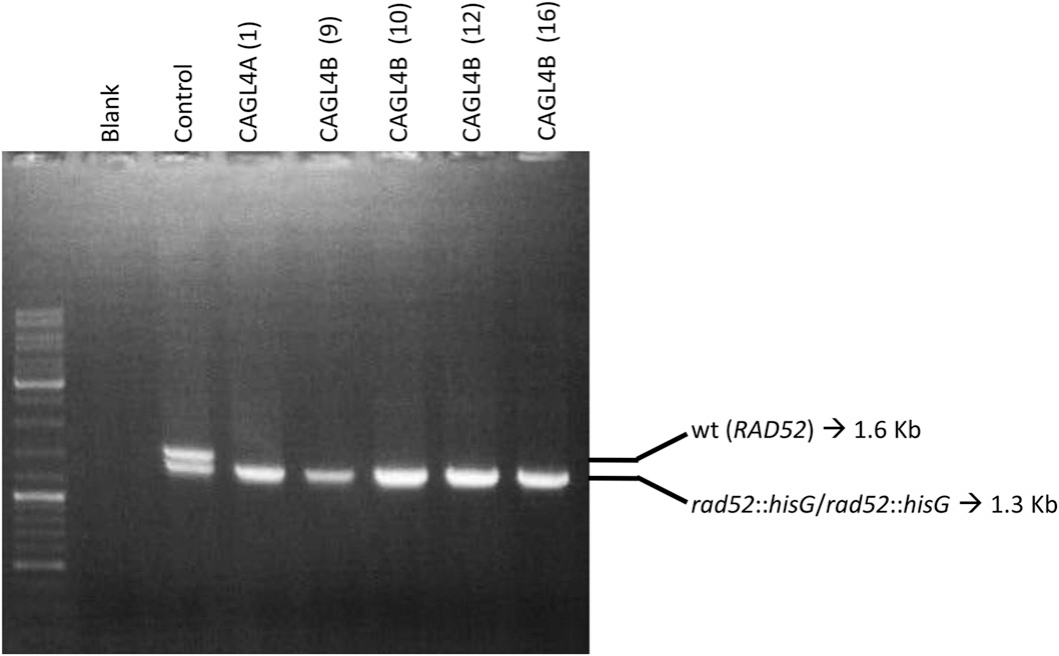
PCR products of *hisG* fragments from *rad52 URA3* pop-out derivatives. Numbers in parentheses identify 5FOA^R^ derivatives for each initial strain.

In contrast to our observations for CAGL4A and CAGL4.1A, chromosome loss was abundant in 5FOA^R^ derivatives from strain CAGL4B: 6/20 (30%) showed LOH of all three SNP markers (Tables 1 and S3). All other derivatives (14/20; 70%) remained heterozygous for at least one SNP marker. CHEF Southerns using a *COX12* probe showed that 71% (10/14) of them have Chr6-derived SNCs larger than 813 kb (Tables 1 and S3; Figs. S8 and S9). Importantly, as shown for strain CAGL4.1A-1, the remaining four CAGL4B derivatives did not show Chr6 size changes and remained heterozygous for all three SNP markers (Fig. S8B, lanes 9, 10, 12 and 16). In addition, PCR exclusively amplified the *hisG* repeat suggesting *URA3* deletion (Figure 5). Furthermore, and consistent with the heterozygosity of SNP makers, Chr6 bands were brighter and broader on CHEF Southern blots, as expected when two homologs with slightly different sizes are present, compared to less bright, single Chr6 homologs that remain after chromosome loss or truncation (*i.e.*, compare lanes 1 and 2 in Figure 4C). These observations support the possibility that in contrast to *S. cerevisiae* (Haber and Hearn 1985; Ozenberger and Roeder 1991; Jablonovich *et al.* 1999; Paques and Haber 1999; Sugawara *et al.* 2000), homology-dependent recombination (SSA or interhomolog recombination) using repeats < 2 kb may occur at measurable rates (10^−6^ - 10^−7^/cell generation) in *rad52 C. albicans* strains. However, the frequency of these events was significantly lower than in wild type (*P* = 0.00005).

Although the absence of either Rad59 or Lig4 in wild type cells did not alter the ratio of *URA3* pop-out *vs.* interhomolog recombination (Table 1), one could argue that either protein could have facilitated the putative *URA3* pop-outs among 5FOA^R^ derivatives in *rad52* strains (Figures 4 and S8). Two independent *rad52 rad59* double mutants (CAGL5A and CAGL5B) (Table 1) exhibited similar frequencies of chromosome loss, chromosome truncation and *URA3* pop-out compared to single *rad52* mutants (CAGL4A/4.1A and CAGL4B) (*P* = 0.57 for *URA3* pop-outs), with chromosome loss only in 5FOA^R^ derivatives from CAGL5B (Table 1, Fig. S10). In addition, the sizes of Chr6 SNCs were similar (compare Figures 4 and S8 with Fig. S10; see also Fig. S9). Overall, and similar to 5FOA^R^ *rad52* derivatives, *URA3* pop-out strains 1) did not show SNCs, 2) remained heterozygous for all three SNPs, 3) conserved both Chr6 homologs (snp132 heterozygous and broader Chr6 bands on PFGE/Southern blots for most of them), and 4) PCR confirmed the presence of an unaltered *hisG* module and the absence of *URA3* (Table S3).

Similarly, the analysis of 5FOA^R^ derivatives from strain CAGL6B (*lig4 rad52A*::*hisG*/*rad52B*::URA-Blaster) (Andaluz *et al.* 2001a) indicated that although most derivatives underwent chromosome loss (17/44; 39%) and chromosome truncation (23/44; 52%) (Tables 1 and S3, Fig. S11), four of them were likely formed by homology-mediated recombination because they satisfied the same four criteria used above for the characterization of *rad52* and *rad52 rad59 URA3* pop-out derivatives. We conclude that *URA3* deletions in *rad52* strains may occur in the absence of either Rad59 or Lig4.

### Rad52-independent spontaneous recombination result from single strand annealing

In *S. cerevisiae*, Rad52-independent SSA requires repeats longer than 2 kb (Haber and Hearn 1985; Ozenberger and Roeder 1991; Paques and Haber 1999; Sugawara *et al.* 2000). However, spontaneous Rad52-independent interhomolog recombination has been reported (Haber and Hearn 1985; Coïc *et al.* 2008). If this is true for *C. albicans*, the presence of *hisG* in a significant fraction of recombinants derived from *rad52* strains cannot be attributed exclusively to SSA. Rather, given the allelic configuration of the wild type strains (*rad52*::*hisG*/*rad52*::URA-Blaster), the *rad52*::*hisG* allele in 5FOA^R^ derivatives could have arisen via conversion of the *rad52*::*hisG-URA3-hisG* allele to *rad52*::*hisG* (Coïc *et al.* 2008). To further investigate this, we constructed new *rad52* strains carrying the *SAT1*-cassette which confers resistance to nourseothricin and then recycled *SAT1* to generate FRT strains (Figure 1, right side, lower branches and Fig. S1; see also Material and Methods) (Reuss *et al.* 2004). Two *rad52* FRT strains, CAGL4A-FRT and CAGL4B-FRT, showed *URA3* loss rates 4- and twofold higher than their *hisG* isogenic counterparts (Fig. S5) and, more importantly, their 5FOA^R^ derivatives contained the *rad52*::*hisG* allele (SSA) at frequencies of 25% (5/20) and 10% (2/20), respectively. The remaining events included chromosome loss (45%) and chromosome truncation (45%) for CAGL4B-FRT derivatives and exclusively chromosome truncations (75%) for CAGL4A-FRT derivatives (Tables 1 and S3).

Deletion of either Lig4 or Rad59 in the *rad52* FRT background did not alter *URA3* loss rates compared to *hisG* strains or precluded the occurrence of SSA. As shown in Tables 1 and S3, SSA was detectable for two independent *rad59 rad52* strains (CAGL5B**-FRT (3/20) and CAGL5B-FRT (5/20)). In addition, chromosome loss and chromosome truncation were also observed (4/20 and 8/20 for CAGL5B**-FRT, and 13/20 and 7/20 for CAGL5B-FRT respectively). Similarly, one SSA-derivative was observed in a *lig4 rad52* FRT strain (CAGL6B-FRT), (1/20, 5%) (Tables 1 and S3). Additional events included chromosome loss (55%; 11/20) and chromosome truncation (40%; 8/20) as expected from the tester allele (Tables 1 and S3). Overall, our results unambiguously demonstrate that an SSA-like process led to the generation of *hisG* from the URA-Blaster in strains lacking *RAD52*, and that this is also independent of Rad59 and Lig4.

### In RAD52 strains, most SSA events are Rad51-independent

*URA3* pop-outs/deletions generally result from intra-chromatid recombination either via SSA-like mechanisms, intra-chromatid crossover or unequal sister chromatid exchange. Importantly, whereas both mechanisms require strand invasion and, therefore, are Rad51-dependent, SSA-like events are independent of, if not inhibited by, Rad51 (Ivanov *et al.* 1996; Jablonovich *et al.* 1999; Pannunzio *et al.* 2008; Manthey and Bailis 2010; Symington *et al.* 2014).

**Figure 6:**
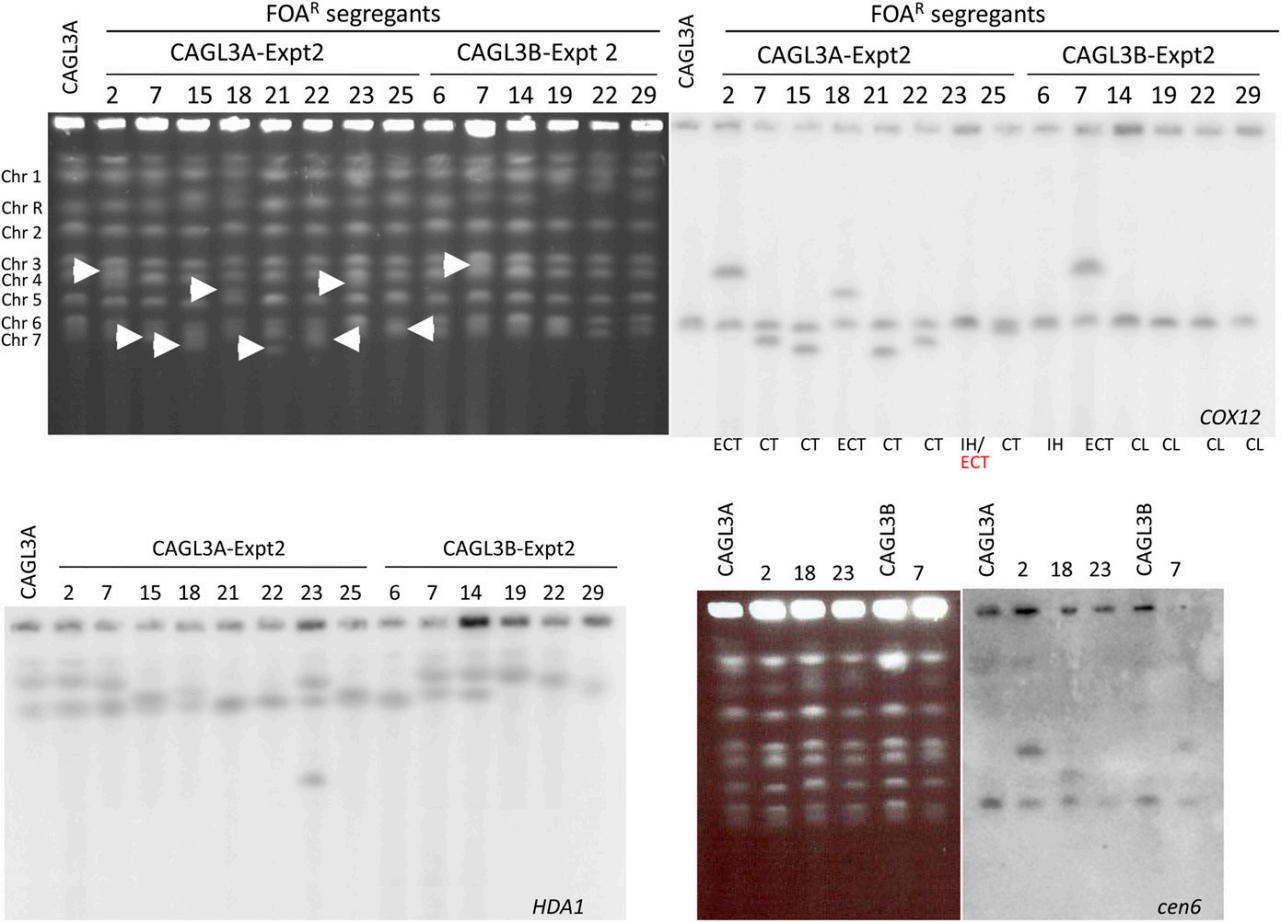
Karyotypes (top-left) of 5FOA^R^ derivatives from strains CAGL3A (test Chr6A) and CAGL3B (test Chr6B). Only derivatives that did not undergo *URA3* loss via pop-out from Expt 2 are shown (see Table S3). Chromosomes were transferred to nitrocellulose paper and hybridized to the *COX12* probe (Chr6) (top-right). Nitrocellulose was stripped and re-probed with the *HDA1* probe (ChrR) (bottom left). SNCs are indicated with white arrowheads. Events accompanying 5FOA^R^ are indicated at the bottom of the top-right panel. Strains 23 (CAGL3A-derivative) and 6 (CAGL3B-derivative) were likely formed by interhomolog recombination involving Chr6; the former also shows an ectopic translocation event involving ChrR (see Fig. S12) (for a summary of the SNP-RFLP analysis see Table S3). Strains showing SNCs larger than Chr6 when probed with *COX12* and *HDA1* (2, 18, 23 from CAGL3A and 7 from CAGL3B) were further analyzed by CHEF Southern using a *cen6* probe. Only derivatives 2, 18 and 7 carried centromeric fragments from Chr6 (bottom right).

To test *RAD51* dependency of the observed *URA3* pop-outs, we deleted *RAD51* in the wild type strain background (CAGL3A and CAGL3B) (Table S1) and found that most CAGL3A 5FOA^R^ derivatives still arose via pop-out (35/50) albeit at a decreased frequency (70%) compared to wild type (>90%). The remaining 5FOA^R^ derivatives (15/50, 30%) retained *RAD52* only. Of these, four strains likely underwent interhomolog recombination (Tables 1 and S3). CHEF Southerns with a *COX12* probe failed to identify Chr6 SNCs among the 5FOA^R^ derivatives from *URA3* pop-outs (not shown). In contrast, in derivatives that retained *RAD52* only, truncations were abundant with SNCs ranging in size from 813 kb to > 850 kb (Figures 6 and S12 and Table S3).

For strain CAGL3B, 76% (38/50) of 5FOA^R^ derivatives arose from *URA3* pop-outs, whereas only two retained *RAD52* and remained heterozygous at all 3 SNPs suggesting that they arose via interhomolog recombination (GC or XO/BIR) near the *RAD52* locus (Table 1). In contrast to CAGL3A derivatives, Chr6 truncations were not detected and chromosome loss was significantly more frequent (10/50; 20%) (Tables 3 and S3, Figure 6).

Importantly, CHEF Southerns with a *COX12* probe revealed novel SNCs with sizes larger than Chr6 for derivatives CAGL3A-2, CAGL3A-18, and CAGL3B-7 (Figure 6, top right). These SNCs also hybridized to a *CEN6* probe (Figure 6, bottom right), and it is therefore likely that these resulted from ectopic translocation involving a centromeric fragment of Chr6 and a fragment of a different chromosome (Figure 6; Table S3). Together, results for 5FOA^R^ derivatives from CAGL3A and CAGL3B show that a similar pop-out bias exists for both tester alleles (Fisher’s exact test, *P* = 0.8299), and that lack of Rad51 significantly decreased the number of the *URA3* pop-outs independent of the allele (*P* = 0.0012, tester allele A; *P* = 0.0337, tester allele B). An interesting consequence of this observation is that, while most pop-out derivatives in the wild type background were generated by SSA, a few may have formed via intra-chromosome crossover or unequal sister chromatid exchange. Furthermore, the absence of Rad51 did not abolish interhomolog recombination since it was still observed among the *RAD52* derivatives.

### The majority of SNCs in rad51 5FOA^R^ derivatives are formed by Chr6 truncation followed by telomere addition

To determine how SNCs larger than wild type Chr6 arose, we tested the possibility that the presence of *hisG* on other chromosomes could serve as translocation hotspot leading to ectopic translocation and the formation of larger chimeric chromosomes. For example, the size of one of the two reciprocal translocation products involving the *hisG* repeats of *rad52*::*hisG* (Chr6) and *rad51*::*hisG* (ChrR, at ∼485 kb) would be ∼1.4 Mb (900 kb from Chr6 plus 485 kb from ChrR), which is close to the size calculated for the ectopic translocation chromosomal bands of 5FOA^R^ derivatives CAGL3A-2, CAGL3A-18, and CAGL3B-7. However, while primers flanking the *RAD52* ORF or the *RAD51* ORF amplified the expected fragments, PCRs with mixed primer pairs did not amplify any products suggesting that ectopic translocations did not involve *hisG* repeats. In addition, CHEF Southerns with a *hisG* probe only hybridized to ChrR in these three strains (Fig. S12, suggesting that the *hisG* fragment on Chr6 had been lost. Importantly, an *HDA1* probe from ChrRL (at ∼450 kb) failed to co-hybridize with the *COX12/CEN6* containing SNCs but hybridized to a novel band of ∼1.4Mb in CAGL3A-23 (Figure 6, bottom left), suggesting that this SNC was generated either by an internal deletion on ChrR or by a translocation involving ChrRL and a centric fragment of one of the smaller chromosomes (Chr5, 6, or 7) (see Figure 6, bottom right). Consistent with either possibility, this SNC, although faintly, hybridized to the *hisG* probe (Fig. S12), suggesting that it could carry sequences of the *rad51*::*hisG* allele.

### RAD52 dosage does not affect URA3 loss

The wild type strains used in the above experiments (*RAD52*/*rad52*::*hisG-URA3-hisG*) carry a single copy of *RAD52*. To investigate whether *RAD52* dosage influences the rate and/or distribution of events responsible for the Ura^-^ (5FOA^R^) phenotype, we analyzed derivatives of strain CAGL27 (*SHE9*/*she9*::URA-Blaster) (Fig. S2), which carries two *RAD52* alleles at its native locus (Materials and Methods) We found that the 5FOA^R^ rate (1.02 × 10^−5^ events/cell generation) was on average threefold higher than that calculated for CAGL1A/CAGL1B strains (Figure 3). PCR and SNP marker analysis of 50 independent 5FOA^R^ derivatives showed that 11 (22%) were formed by interhomolog recombination (gene conversion/crossover/BIR), one (2%) by inactivation of *URA3*, and 38 (76%) by URA3 pop-outs (Table 1). Importantly, for strain CAGL28, which has a single copy of *RAD52*, both the 5FOA^R^ rate at the *SHE9* locus (1.2 × 10^−5^ events/cell generation) (Figure 3) and the distribution of events (81% deletions and 19% interhomolog crossover) were similar to strain CAGL27 (Table 1). Therefore, the *RAD5*2 dosage did not seem to affect the SSA-like bias significantly.

## Discussion

In the present study, we aimed to study the mechanisms involved in direct repeat recombination in *C. albicans* wild type strains and recombination mutants. We took advantage of the URA-Blaster (inserted at the *RAD52* locus on Chr6) to determine rates of direct-repeat recombination measured as *URA3* loss (resistance to 5FOA), and we used SNP-RFLP and CHEF-Southern analyses to determine the underlying mechanisms. We found that for the wild type strain (CAI4), *URA3* pop-outs were the major events responsible for 5FOA^R^ whereas other events identified here as interhomolog recombination and *URA3* mutational inactivation were much less frequent and independent of the Chr6 allele examined. Importantly, interhomolog recombination resulted in very long LOH tracts in about 5–10% of 5FOA^R^ derivatives, most of them leading to homozygosis of all SNP markers, which is consistent with BIR or reciprocal crossovers (Forche *et al.* 2011; Cauwood *et al.* 2013; Symington *et al.* 2014). It is worth noting that the major repeat sequence on Chr6 is located between snp123 marker and *cen6* (Figure 1), the region where most interhomolog recombination occurred, suggesting that it could act as a recombination hotspot in wild type cells (Lephart and Magee 2006; Marton *et al.* 2019). The possibility that these strains exhibit phenotypes attributable to off-target effects calls for an exhaustive characterization of *C. albicans* genetically engineered strains involving recombination as recently demonstrated for CAI4 (Ciudad *et al.* 2016).

In the absence of recombination proteins Rad51 or Rad52 chromosome loss and chromosome truncations were frequent, which supports the existence of selective pressure to maintain a complete Chr6A homolog; its loss likely may result in cell death. This conclusion is consistent with previous results indicating that only one homolog of several chromosomes can be lost or is preferentially lost (Andaluz *et al.* 2011; Hickman *et al.* 2013). As a diploid, *C. albicans* may allow the generation of high levels of heterozygosity including the appearance and persistence of recessive lethal alleles of one or more essential genes on one homolog, as was recently shown for Chr4 and Chr7 (Feri *et al.* 2016; Marton *et al.* 2019). Therefore, the nature and relative frequency of events responsible for the loss of *URA3* may depend significantly on the homolog used as tester chromosome.

### Rates of URA3 loss in mutants with defective homologous recombination

We found little variation in *URA3* loss rates for single mutants (including *rad52*) compared to wild type. This is in contrast with the significant decrease in 5-FOA^R^ frequency exhibited by haploid *S. cerevisiae rad52* and, to a lesser extent *rad59*, in a similar assay using *URA3* flanked by 2.4 kb-long repeats (Halas *et al.* 2016). Under these conditions, loss of *URA3* via SSA is drastically decreased (*rad52*) and other mechanisms such as chromosome loss and chromosome truncation may be detrimental. By contrast, a diploid *rad52* strain has the potential to become Ura^-^ by chromosome loss and chromosome truncation. This is particularly true for *C. albicans*, whose genome plasticity is well documented (Rustchenko 2007; Selmecki *et al.* 2009; Forche 2014). Some variation between the *URA3* loss rates of the several strains may also derive from the diploid state of *C. albicans*. For instance, in wild type *S. cerevisiae* variation in *URA3* loss rates from the URA-Blaster inserted at five different loci was intrinsically greater in diploids compared to haploids (Cauwood *et al.* 2013). The validity of our assay is further supported by the observation that *URA3* loss rates were locus- and dosage-independent. The significant increase in *URA3* loss rate in *rad52 rad59* and *rad52 lig4* double mutants compared to *rad52* single mutants (5.5 and 7.eightfold, respectively) may simply suggest that Rad59 and Lig4 are suppressing the formation of lesions that form in a *rad52* background, most likely because they are repairing these lesions. However, regardless of the mechanism(s) involved, it may stem from the additive effect on genetic instability caused by the lack of more than one gene.

### In Rad52^+^ cells, URA3 pop-outs are Rad59-independent and occur through an SSA-Like mechanism

The absence of Rad59 did not alter the frequency of SSA in *C. albicans*. This result contrasts with previous work in *S. cerevisiae* showing that depletion of Rad59 significantly decreased the formation of the SSA product and the number of survivors regardless of repeat length (Sugawara and Haber 1992; Sugawara *et al.* 2000). An important difference between both systems is that the *S. cerevisiae* study (Sugawara *et al.* 2000) used a haploid strain and created a DSB between the repeats whereas we have determined spontaneous recombination in a diploid cell. However, other studies in diploid *S. cerevisiae* strains have found that Rad59 is also required for spontaneous SSA-like events of short repeats (Halas *et al.* 2016). It will be interesting to determine whether direct repeats shorter than *hisG* (1.1 kb) would affect *URA3* loss in the absence of CaRad59.

It should be noted that, unlike wild type, the *rad59*::*hisG* alleles (Chr4) in *rad59* strains could represent potential sites for ectopic translocation involving sequences of the *rad52*::*hisG-URA3-hisG* allele (Chr6). We think this is unlikely because translocations involving Chr6 were not observed among the 84 5FOA^R^ derivatives in the *rad59*-ΔΔ strain background. However, it is possible that *hisG*-mediated ectopic translocations may occur at rates below the limit of detection. In *S. cerevisiae*, for example, spontaneous ectopic recombination between Ty1 interspersed direct repeats occurred at a much lower rate (10^−8^/cell generation, respectively), which is below the threshold of detection in the system used here (Chan and Kolodner 2011).

### Deletion of CaRAD51 does not abolish interhomolog recombination, reduces SSA frequency, and induces ectopic translocation, chromosome loss and chromosome truncation

In *S. cerevisiae*, SSA is Rad51-independent whereas crossovers are dependent on Rad51. We found that depletion of Rad51 caused a statistically significant drop in the rates of *URA3* pop-outs. This is in striking contrast to a reported increase of SSA between direct repeats in *rad51* null mutants of haploid *S. cerevisiae* (McDonald and Rothstein 1994; Bai and Symington 1996; Ivanov *et al.* 1996; Jablonovich *et al.* 1999; Sugawara *et al.* 2000; Pannunzio *et al.* 2010; Halas *et al.* 2016), in *rad51* loss-of-function mutants in mammalian cells (Stark *et al.* 2004), and for Rad51 inhibition of Rad52-mediated annealing of complementary ssDNA *in vitro* (Wu *et al.* 2008). The decreased frequency of SSA in the absence of Rad51 suggests that although most *URA3* pop-outs in wild type *C. albicans* were due to SSA, a few likely resulted from intra-chromatid crossover. Differences in SSA requirements between both yeasts might arise from the assay conditions (spontaneous in *C. albicans vs.* mostly DSB-induced in *S. cerevisiae*), the ploidy of the strains analyzed, or a differential regulation of SSA/intra-chromatid crossovers that could have evolved in response to the higher number of repeats in *C. albicans* or other species-specific traits.

**Figure 7:**
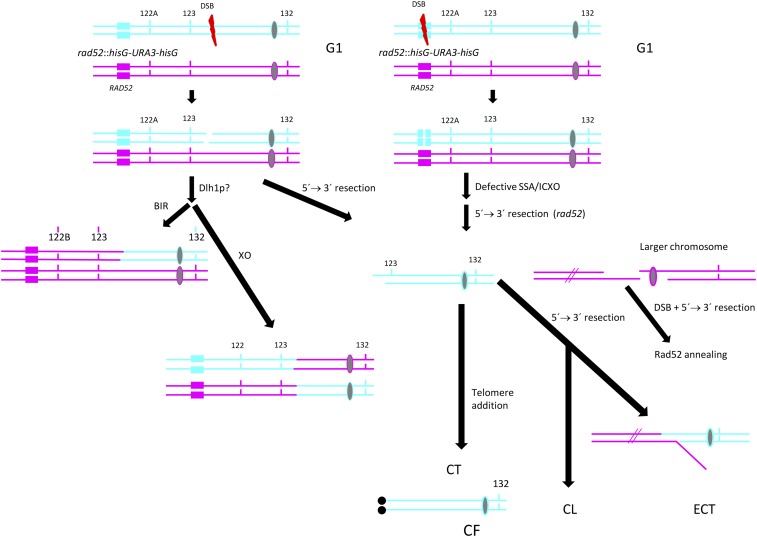
Model for genetic events in *rad51* mutants. Left. Spontaneous (likely *DLH1*-mediated) recombination (crossover/BIR) between Chr6 homologs in G1 followed by the occurrence of a DSB. Each line accounts for ssDNA. Right. A DSB within the cassette region (fragile site) is followed by resection. Telomere addition at the resected end results in SNC formation (see Discussion). In *rad51* strains ectopic translocation can occur if a complementary ssDNA tailed duplex of another broken and resected chromosome (red lines) is detected using the annealing activity of Rad52. If resection continues and trespasses a threshold (which we have traced to the neighborhood of SNP123) the Chr6 fragment cannot be maintained in the absence of Rad52 (and perhaps Rad51) resulting in chromosome loss (loss of Chr6B) or cell dead (loss of Chr6A).

A second interesting finding was that depletion of Rad51 did not abolish interhomolog recombination, which could still be caused by Rad51-independent BIR (VanHulle *et al.* 2007) or, more likely, by true crossover/BIR catalyzed by the Rad51-paralog *DLH1* (Figure 7). *DLH1* is the ortholog of the meiotic recombinase *DMC1* that mediates strand invasion in *S. cerevisiae* meiotic cells (Bishop *et al.* 1992; Diener and Fink 1996). It is clear, however, that an important fraction of the events requiring strand invasion (inter-homolog, inter-sister chromatid, or intra-chromatid crossovers) initiated in the absence of Rad51 are defective and channeled toward ectopic translocation, chromosome loss and chromosome truncation (Table 1, Figure 7). Importantly, whereas ectopic translocations were completely absent in the presence of Rad51, they occurred at rates of 1.5 × 10^−7^ events/cell generation in *rad51* strains and likely involved endogenous homologous sequences of another chromosome, but not *hisG* repeats (Figure 6), further supporting the idea that the latter are not hotspots for translocation.

We have previously shown that centric fragments of truncated chromosomes observed in *rad52* strains of *C. albicans* are maintained when sealed by *de novo* telomere addition using junction sequences common to both chromosome and telomere (Andaluz *et al.* 2011). Therefore, it is likely that some resectioning of DSBs occurs before bases complementary to telomere repeats are exposed. Similarly, resectioning of DSBs can also expose ssDNA tracts complementary to sequences present on a different chromosome (Figure 7). In this scenario the absence of Rad51 would increase the substrate pool for translocation mediated by *RAD52*/*RAD9*-dependent SSA, consistent with the absence of translocations in *rad52* mutants. We conclude that Rad51 is a strong suppressor of spontaneous ectopic translocation in *C. albicans*. This is consistent with findings in *rad51* haploid *S. cerevisiae* where spontaneous Ty1-mediated GCR rates were increased sevenfold (Chan and Kolodner 2011) and with the spontaneous ectopic translocation frequency between 300 bp of identical sequence in diploids (Pannunzio *et al.* 2008; Manthey and Bailis 2010). In the latter case, the rate of translocation for Sc*rad51* strains was 1.1 × 10^−7^/cell generation, which is similar to what we found for Ca*rad51* in our study.

### On the generation of SNCs in the absence of CaRad52

Our assay did not select for *rad52* 5FOA^R^ derivatives carrying a centromeric SNC that retains a functional *URA3*. Given the size of Chr6 (1032 kb) and the distance between *rad52*::*hisG-URA3-hisG* and the left telomere (≈ 95 kb), the maximum expected size for a SNC is 940 kb, which is consistent with the observation that SNCs were always smaller than 945 kb (the size of Chr7). However, there are no constraints for the minimal size of a Chr6 SNC other than retention of *cen6*, which is only 53 kb away from the right telomere. In fact, a 95 kb centromeric fragment was conserved following truncation of Chr6 *in vivo* (Baum *et al.* 2006). The large size of SNCs (usually > 813 kb and never smaller than 666 kb), could stem from the inability of *rad52* cells to maintain SNCs smaller than 500 - 600 kb (high chromosome loss frequency). In agreement with this, it was shown previously that the only Chr6-derived SNC identified among spontaneous histidine auxotrophs generated by *rad52* cells was ≈ 600 kb (Andaluz *et al.* 2011), which matches the size of the smallest SNCs detected in this study (≈ 630 kb). One explanation for the abundance of large SNCs is the presence of a fragile site between snp122 and the left telomere whose tendency to break increases during DNA replication in the absence of Rad52 and, to a lesser extent, Rad51. Importantly, in *rad52* or *rad51* haploid *S. cerevisiae*, defective fork restart at damaged (methylated) sites results in chromosome breakage and cell death (González-Prieto *et al.* 2013), and a similar defect could generate SNCs in diploid *C. albicans rad52* and *rad51* derivatives. An attractive possibility is that the URA-Blaster acts as a fragile site due to unusual DNA or chromatin structure that converts it into a locus difficult to replicate and thereby mimicking DNA damaged sites. To account for the actual size of SNCs, the initial break at the URA-Blaster would require a minimum of 100 - 120 kb resection which, according to data from *S. cerevisiae*, could be too long for wild type (Zhu *et al.* 2008) but not for *rad52* strains (Sugawara and Haber 1992). We do not rule out that the *C. albicans* major repeat sequence also may act as a recombination hotspot (Lephart and Magee 2006; Chibana and Magee 2009; Marton *et al.* 2019) and become a breakage site in the absence of homologous recombination proteins. However, if that were the case, resulting SNCs would be too small to be maintained in *rad52* strains.

In this study we show that although recombination pathways are basically conserved, *C. albicans* exhibits specific requirements for mitotic recombination that affect expansion and contractions of repeated sequences, including Rad52-independent SSA (for repeat lengths as short as 1.2 kb) and Rad51-independent interhomolog recombination. This opens up the exciting possibility that, in addition to affecting genome structure, specific features of the recombination machinery may have evolved to facilitate variation. Ongoing and future research will be studying the impact of specific stresses on repeat stability and identifying recombination requirements for repeats of reduced length.
